# Silicon-Carbon composite anodes from industrial battery grade silicon

**DOI:** 10.1038/s41598-019-51324-4

**Published:** 2019-10-15

**Authors:** Hanne Flåten Andersen, Carl Erik Lie Foss, Jorunn Voje, Ragnar Tronstad, Tommy Mokkelbost, Per Erik Vullum, Asbjørn Ulvestad, Martin Kirkengen, Jan Petter Mæhlen

**Affiliations:** 1 Institute for Energy Technology, P.O. Box 40, NO-2027 Kjeller, Norway; 2 Elkem, Oslo, Norway; 3 SINTEF, Trondheim, Norway; 4 Department of Physics, University of Oslo, Oslo, Norway; 5 Kjeller University Graduate Center (UNIK), Kjeller, Norway

**Keywords:** Batteries, Batteries

## Abstract

In this work, silicon/carbon composites for anode electrodes of Li-ion batteries are prepared from Elkem’s Silgrain® line. Gentle ball milling is used to reduce particle size of Silgrain, and the resulting Si powder consists of micrometic Si with some impurities. Silicon/carbon composite with CMC/SBR as a dual binder can achieve more than 1200 cycles with a capacity of 1000 mAh g^−1^ of Si. This excellent electrochemical performance can be attributed to the use of a buffer as a solvent to control the pH of the electrode slurry, and hence the bonding properties of the binder to the silicon particles. In addition, the use of FEC as an electrolyte additive is greatly contributing to a stabilized cycling by creating a more robust SEI layer. This work clearly demonstrates the potential of industrial battery grade silicon from Elkem.

## Introduction

When the solar industry started, it consistently used scrap silicon from failed semiconductor silicon production facilities. As demand grew, solar grade silicon became a separate product category with slightly less demanding quality standards, but significantly lower cost, than semiconductor grade silicon. Now, with the introduction of silicon in battery anodes, this idea is brought up again, and it has been proposed to make silicon for batteries cheaply by using scrap material from solar silicon production.

Silicon has recently been proposed as one of the most promising anode materials for lithium-ion batteries due to its high theoretical lithium storage capacity (3579 mAh g^−1^ for Li_15_Si_4_)^1^, a great improvement to that of commercial graphite anodes (372 mAh g^−1^ for LiC_6_)^2^, high volumetric capacity, a relatively low discharge voltage (the average delithiation voltage of Si is 0.4 V)^3^ and low cost^4,5^. However, the process of commercializing silicon anode is not straightforward due to major drawbacks such as volume expansion of the silicon upon Li insertion^1,6–8^. The formation of Li_x_Si alloys causes cracking and pulverization of Si particles as well as cracking and adhesion problems in the Si-film electrode^9,10^. This in turn leads to the loss of electrical contact and a short cycle life.

In addition to the pulverization of the silicon particles, the cracking of active material grains may expose new anode surface to electrolyte and induce formation of a fresh solid electrolyte interphase (SEI)^11,12^. Subsequently, this leads to low coulombic efficiency and increased electrode resistance as well as electrolyte depletion. Thus, the stability of the SEI is crucial for the functioning of the electrode^8,13^.

To improve the stability of the silicon anode and meet the challenges mentioned above, several structural design measures can be implemented. The material stress during lithiation can be minimized by nanostructuring electrodes with enough free space volume to accommodate the expansion. Generations of nanostructures, such as nanowires^14,15^, nanofibers^16,17^, thin films^18,19^, core-shell^20,21^, hollow Si with and without clamping^14,22,23^ have been investigated and proved interesting due to improved cycle life, high-surface-to-volume ratios which can facilitate efficient transfer of lithium^24^. Although nanomaterial designs have resolved some major problems and extended the cycle life, other challenges still exist regarding nanostructured electrodes. For example, the high surface area of nanostructured electrodes might present a challenge for limiting irreversible capacity caused by the formation of the SEI layer. As mentioned by Lin *et al*.^25^, reducing the size scale gives a rise to low tap density and high interparticle resistance due to more space in between particles and higher surface area. This results in an electrode with low volumetric capacity, meaning one would have to manufacture a thicker electrode, with longer electron pathway, to achieve the same mass loading as an electrode with a higher tap density. In addition, several of these nanostructures and morphologies involve an extensive and rather costly production step, which make them irrelevant to silicon production on an industrial scale^26^. While silicon anodes are better than graphite anodes in terms of capacity, it is necessary that the production methods are comparable in cost to make silicon anodes a viable candidate to compete with graphite anodes commercially.

A popular strategy to cope with the unstable SEI layer is the use of additives to the electrolyte. Additives such as vinylene carbonate (VC) and fluoroethylene carbonate (FEC) are known to favour a denser and more stable SEI layer, and thus increasing the cycle life^27–29^. Another approach to control the large expansion of silicon upon lithiation, is to cycle electrodes to less than full capacity^10,30,31^. For half cells this if often achieved using capacity constrained cycling methodologies (shallow depth of charge or discharge) that enhances the lifetime of the Si anodes by retarding mechanical degradation of the material^32^. In a full cell configuration, the silicon anode is paired with a cathode with a lower specific capacity. When the cell is charged to the voltage limit, a fixed amount of lithium is transferred to the anode. Usually, the loading of the cathode is enough to allow for a reversible capacity of 1000 mAh g^−1 ^^30^ for the silicon anode. Even though the capacity of the silicon anode has the potential to store ten times more capacity than graphite, further improvements in the energy density of the battery is limited by the cathode capacity. The main reason for this is the necessity of matching the capacity of the anode with a cathode which is far less energy dense. It is therefore also important to develop high capacity cathodes to unlock the true potential of silicon anodes^33^.

In this work, we aim to use industrial scale silicon from Elkem in a composite material as a negative anode for the lithium-ion battery and achieve a considerable improvement in capacity to commercial graphite anodes, while maintaining cycle stability. To reach this goal, we looked for: (i) suitable mechanical processing methods to reduce particle size, (ii) bonding properties of silicon-carbon-binder material, (iii) the influence of electrolyte additives on the stability of the SEI layer and (iv) constrained cycling methods to control the silicon expansion.

## Material and Methods

### Material preparation

Silgrain® (0–10 µm, Elkem) was used as a silicon source. The particles size distribution was measured by laser diffraction (Malvern Particle Size Analyzer) and the purity of Silgrain was measured using XRF. The Silgrain® was produced at Elkem Bremanger via a hydro-metallurgical leaching process. To reduce the particle size ball milling of the larger silicon grains was performed (Fritsch Pulverisette). The milling time was varied between 5 and 180 minutes, while the milling speed was kept constant (800 rpm). A milling vial of ZrO was used and the ball to powder ratio was 4:1 with 10 mm sized ZrO-balls.

TEM measurements were performed with a double C_s_ corrected cold field emission gun JEOL JEM-ARM200F operated at 200 kV. Powder samples were directly dispersed onto carbon coated Cu TEM grids.

X-ray diffraction (XRD) were carried out in a Bruker D8 Discover X-ray diffractometer, to estimate crystallite sizes by whole-profile fitting the data to be ca 85, 50, and 45 nm, respectively (peak broadening was assumed to be only due to particle size, no strain-corrections were applied).

### Electrode preparation

Composite electrodes were made of the resulting silicon powder (0–10 µm) together with carbon black (Super C 65, Timcal), graphite (SLP30, Timcal) and carboxymethyl cellulose (CMC, M_w_ = 90 000, DS = 0.7, Sigma-Aldrich) as a binder. The ratio between the components was 60:15:10:15, unless otherwise specified. For some experiments a second binder, styrene-butadiene rubber, (SBR, PSBR100, Targray) was added to the composite system to examine the effect of a dual binder. A buffer solution with pH 3 consisting of citric acid (Sigma-Aldrich) and KOH (Sigma-Aldrich) was used as a solvent. Initially, the binder material and the solvent were mixed, and the binder dissolved via ultra-sonication. After mixing for at least 30 minutes, the remaining components were added, and the solution was mixed with an UltraTurrax dispersing instrument for 30 minutes at 6000 rpm. The slurry was tape casted onto a 16 µm structured copper foil where the wet thickness of the slurry could be adjusted. The composite tape was dried for 20 h at room temperature, and then for 3 h at 120 °C under vacuum.

### Electrochemical testing

Half cells were prepared with 15 mm disks of the silicon/carbon composite as the working electrode in 2032 coin cells. A lithium metal disk was used as the counter electrode, with Celgard separators (CG 3401) and LP30 electrolyte (1 M LiPF6 solution in dimethyl carbonate-ethylene carbonate (1:1)). For some experiments 10 wt% fluoroethylene carbonate (FEC) was added to the electrolyte. The cells were assembled in a glove box under argon atmosphere. Cell cycling was performed at room temperature with an Arbin Battery tester. The cells were cycled with 3 initial formation cycles at C/20 followed by cycling at C/10, unless otherwise specified. The voltage range was 0.05–1 V vs. Li/Li^+^.

## Results and Discussion

### Silicon and composite characterization

The elemental analysis of Silgrain® as a starting material is shown in Table 1. As Silgrain® is a metallurgical grade of Si, some impurities are expected, and traces of Fe, Al, Ca and Ti have been found in the powder. Pure silicon is known to be highly resistive; however, silicon with impurities is expected to behave more like a semiconductor. It is not clear whether the impurities have a detrimental effect on battery cycling stability and some impurities might improve electron transport for Li^+^ insertion^34^.

**Table 1:** Typical purity of Silgrain®, measured by XRF.

Analysis	Si	Fe	Al	Ca	Ti
	*wt%*	*wt%*	*wt%*	*wt%*	*wt%*
Max		0.05	0.12	0.02	0.005
Min		0.02	0.07	0.005	0.001
Typical	99.7	0.035	0.09	0.01	0.002

**Figure 1:**
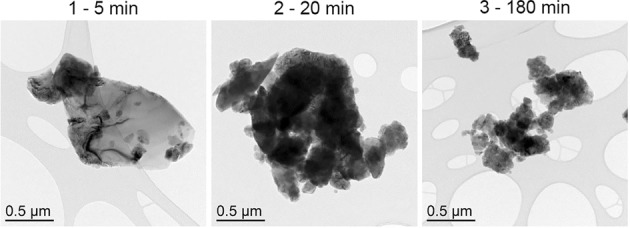
TEM images of Si powder ball milled at 800 rpm for (1) 5 minutes, (2) 20 minutes and (3) 180 minutes.

**Figure 2:**
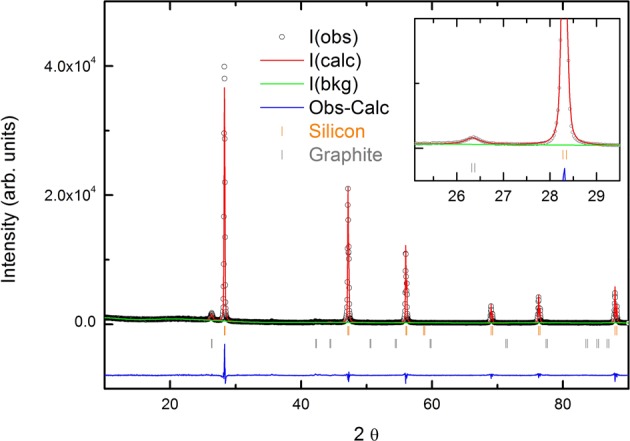
XRD pattern of ball milled Silgrain® milled together with graphite (8:2). I(obs) is the observed measurement. I (calc) is the calculated measurement. I(bkg) is the background, and Obs-Calc is the observed measurement minus the calculated. The inset magnifies the area where we expect to see the graphite peak.

**Figure 3:**
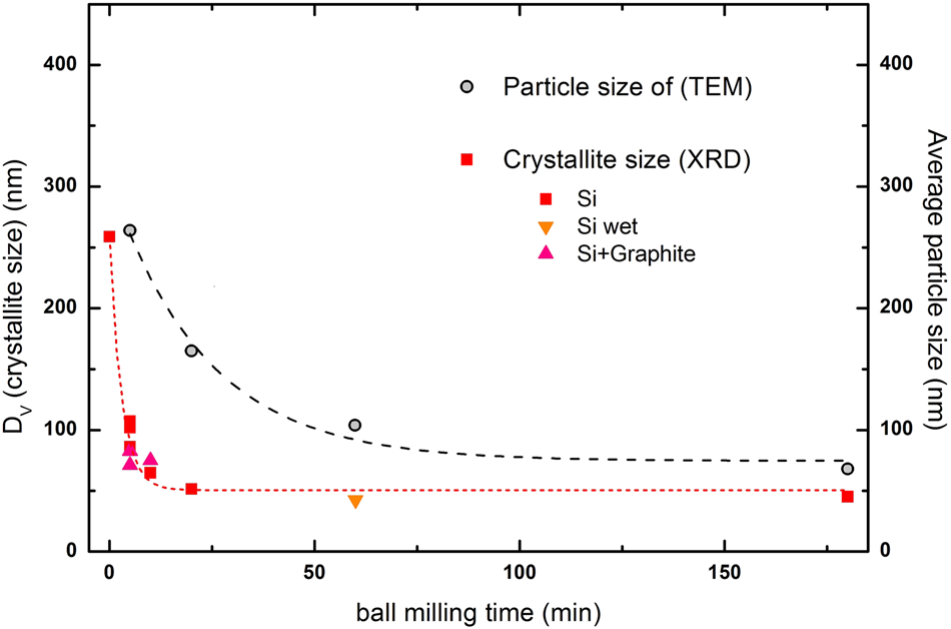
Crystallite and particle size of Silgrain® as a function of ball milling time. Crystallite size determined from XRD measurements, while average particle size was determined by TEM analysis.

Figure 1 shows TEM images of Si powders ball milled for 5,20 and 180 minutes. Most notably, milling time decreases particle size. A typical XRD pattern of Silgrain ball milled together with graphite (8:2 ratio) is shown in Fig. 2. The inset magnifies the area we expect to see the graphite peak, which is the small peak between 2 theta values between 26° and 27° before the large peak around 29° attributed to silicon. From the width of these peaks the crystallite size as a function of ball milling is estimated and summarized in Fig. 3, together with the estimated particles sizes from TEM measurements. These all confirm that the crystallite size and the particle size all decrease as a function of milling time. The crystallite size estimated from XRD was performed both in pure silicon and silicon + graphite composite, as well as one wet Si sample, which all follow the same trendline. The effect of milling on crystallite size levels off after about 50 min, which is useful to know as the cost of anode production would increase with milling time.

### Electrochemical behavior

**Figure 4:**
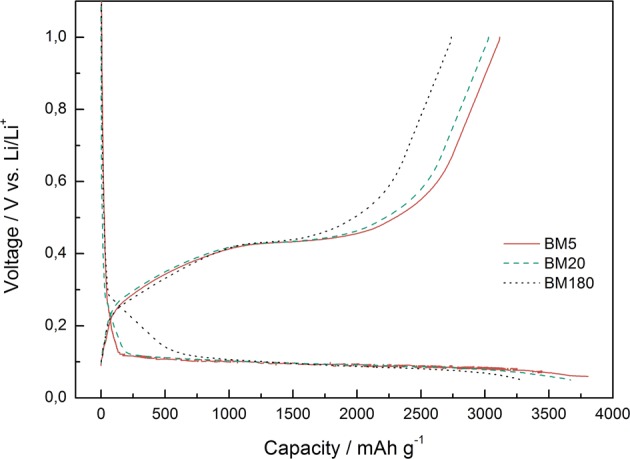
Voltage profiles from first cycle of silicon/carbon composites (C/20 rate) ball milled for 5 (BM5), 20 (BM20) and 180 minutes (BM180). The silicon (60%) was pre-milled before mixed with 30% graphite, 8% binder (CMC) and 2% carbon black.

Silicon/carbon composite electrodes were prepared from silicon which was ball milled 5 minutes (BM5), 20 minutes (BM20) and 180 minutes (BM180). Voltage profiles from these electrodes from the first cycle in Fig. 4 (C/20 rate with LP30 electrolyte) provide an initial insight into the electrochemical properties. The BM5 sample shows the highest discharge and charge capacity of 3801 and 3117 mAh g^−1^, respectively, corresponding to an initial Coulombic efficiency of 82%. The samples ball milled 20 and 180 minutes, show a lower discharge capacity of 3692 and 3287, respectively, with initial Coulombic efficiencies of 82 and 83%.

The discharge curves show a clear difference where BM5 and BM20 rapidly reach a well-defined plateau at ~0.1 V, while the BM180 has a longer slope between 0.3–0.1 V. The flat plateau at 0.1 V is usually observed with Si particles in the micrometer range. In comparison, the voltage curve of the BM180 sample show a variation in discharge without a well-defined plateau which is a behavior commonly observed with nanometric Si particles^35^. The flat plateau (0.1 V) indicates a transformation from c-Si alloy to a-Li_x_Si alloy. The BM180 sample reacts with Li at a higher voltage (~0.3 V) as the energy required to break up the Si-Si bond in the (111) is not required for a-Si^9^.

One of the possible reasons for the lower initial discharge/charge capacity of the Si milled 180 minutes could be explained with a higher content of SiO_x_ originating from higher surface area and longer milling time since a higher content of SiO_x_ corresponds to a lower theoretical capacity. After ball milling, fresh surface area is exposed to ambient atmosphere, and it has previously been seen that this in fact increases the oxygen content^36^. The theoretical reversible capacity for SiO, assuming all Si can be lithiated in SiO, is close to 2680 mAh g^−1^ ^37^, so it is reasonable to assume that this could be a contributing factor. However, this is a complex topic and a recent review on the role of oxygen vacancies for lithium/sodium – ion batteries stated that there is a lack of research and theory on silicon oxide materials^38^.

**Figure 5:**
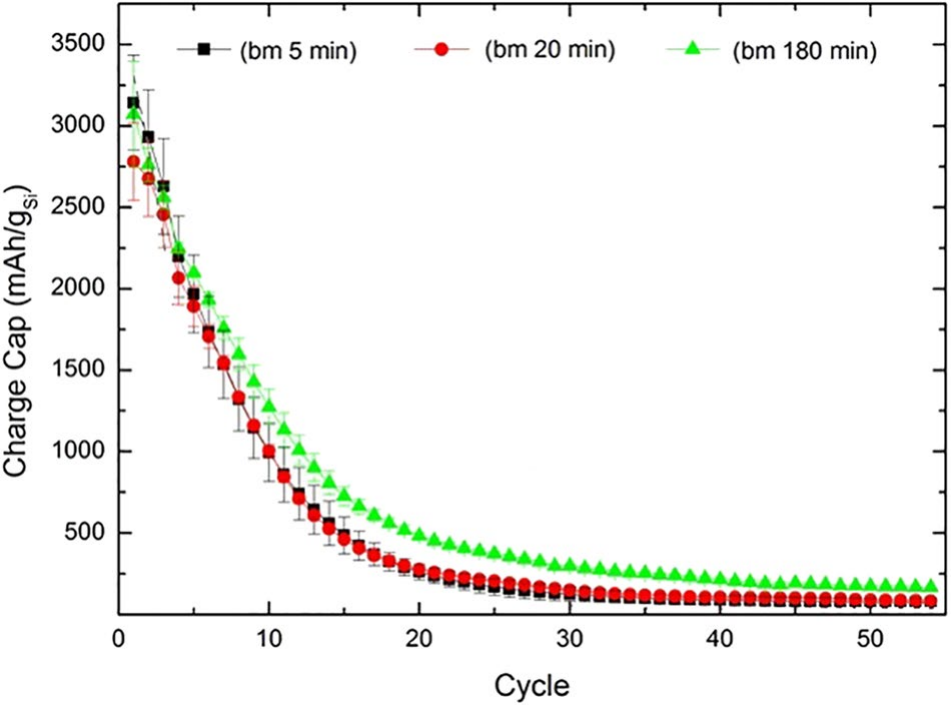
Charge capacities as function cycle number for samples ball milled for 5 (BM5), 20 (BM20) and 180 minutes (BM180).

From Fig. 4 no significant advantage can be seen from using silicon ball-milled 180 minutes, when regarding the initial charge/discharge capacities. Capacities after 10 cycles (Fig. 5) show no clear trend that a longer milled material gives higher cycling stability. However, all samples show a very low capacity after 10 cycles, and only about 40% of the initial capacity remains. This extensive degradation makes further interpretation of the material and the differences challenging.

### The use of buffer solution to control slurry pH

**Figure 6:**
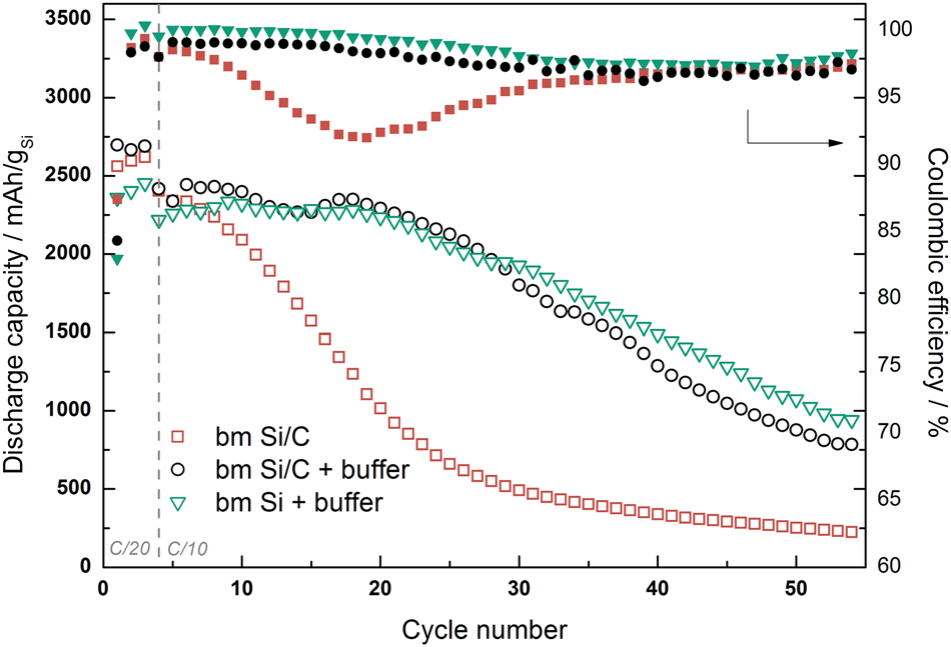
The use of buffer as a solvent to control the pH of the anode slurry. The cells have been cycled between 0.05–1 V at C/20 for the first three cycles, and C/10 rate subsequently.

Literature studies have shown that preparing the slurry in a pH 3 buffer is important to achieve grafting of the CMC binder which can strongly improve the cycle life^10,39,40^. Mazouzi *et al*.^39^ showed that by using a buffer, covalent bonding can be promoted between silicon and the binder. They attributed this to neutralization of SiO^−^ and COO^−^ groups into SiOH and COOH by keeping the pH lower than the isoelectric point of silicon particles (~3.5) and pKa of CMC (3.5). This results in a slurry where esterification is heavily favored upon drying^40^, thus yielding a higher bonding strength due to covalent bonding. Evidence of this can be found from the cycle performance and the coulombic efficiency in Fig. 6. Si/C composite electrodes were prepared from ball milled starting material. Silicon was either ball milled 5 minutes alone, or 80% Si was ball milled with 20% graphite. Both ball milled samples were mixed with carbon (30% carbon as total amount) and 10% binder. The two composite types where tested with and without a buffer solution as the solvent. Cells were prepared and cycled between 1.00–0.05 V vs. Li/Li+. The initial C-rate was kept at C/20 and changed to C/10 after 3 cycles. The initial capacity is 2560 mAh g^−1^ for bm Si/C without the buffer solution and 2700 mAh g^−1^ and 2360 mAh g^−1^ for the bm Si/C and bm Si with buffer solution, respectively. However, after only 10 cycles, the difference in discharge capacity has changed in favor of the cells prepared with buffer solution. By controlling the pH of the slurry, the discharge capacity is kept at a higher level for 20 cycles before a degradation is observed. The sample without pH control experiences a sudden capacity decrease, which is also reflected in the very low coulombic efficiency observed in the first 20 cycles. The low coulombic efficiency for all samples in the first cycle could be attributed to irreversible reactions, such as SEI formation.

### Binder system

The role of binders is very critical for Si/C electrodes to maintain the electrode structure and obtain sufficient cycle stability. Most studies have focused on the use of poly vinylidene fluroride (PVDF) and carboxymethyl cellulose (CMC). The more commercial binder, used in graphite anodes, PVDF, fails to accommodate the large changes in volume for Si anodes and becomes unable to keep the particles together, which is essential to maintain good electrical conductivity^40^. The CMC binder typically displays a better performance and is often the better choice for Si-based anodes. However, recent studies have reported an improved cycle life by using an aqueous binder containing both CMC and the elastomeric styrene butadiene rubber (SBR)^13,41^. CMC is a polymeric derivate of cellulose containing carboxylate anion and hydroxyl functional groups. These groups make CMC and a very good thickening agent and soluble in water. Since SBR is an elastomer it has a high flexibility, but also strong binding properties and it is often used to enhance the adhesion force between the film and the current collector^42,43^. The SBR/CMC binder has shown great potential for silicon-based materials.

**Figure 7:**
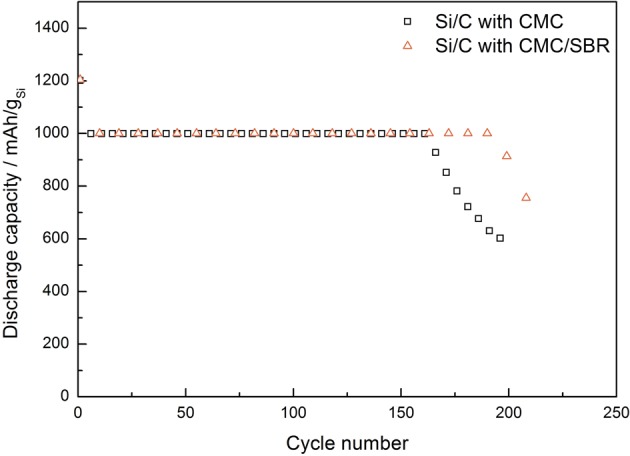
Cycling of discharge capacities of Si/C composite with various binder systems, cycled with a capacity limitation at 1000 mAh g^−1^ of Si in LP30 electrolyte.

Figure 7 shows the discharge capacities of the electrodes prepared with different binders. The cells were cycled with a limited charge capacity. A limited capacity regime can improve the cycle life by reducing the strain on the lattice during lithiation.

While not utilizing the full potential of the silicon anode, the capacity of a shallow charged Si anode can be as high or higher as traditional carbon anode, and the cycle life can be greatly improved^32^. The cells in Fig. 7 were limited to a discharge capacity of 1000 mAh g^−1^ per silicon (after the first cycle), which represents 600 mAh g^−1^ of the total electrode, a discharge capacity higher than commercial graphite anodes can achieve. The Si/C-CMC cell obtained 161 cycles at the set capacity before degradation commenced, while the Si/C-CMC/SBR cell was able to cycle longer (190 cycles) at the set capacity.

### The use of electrolyte additives to control SEI formation and improve cycling stability

**Figure 8:**
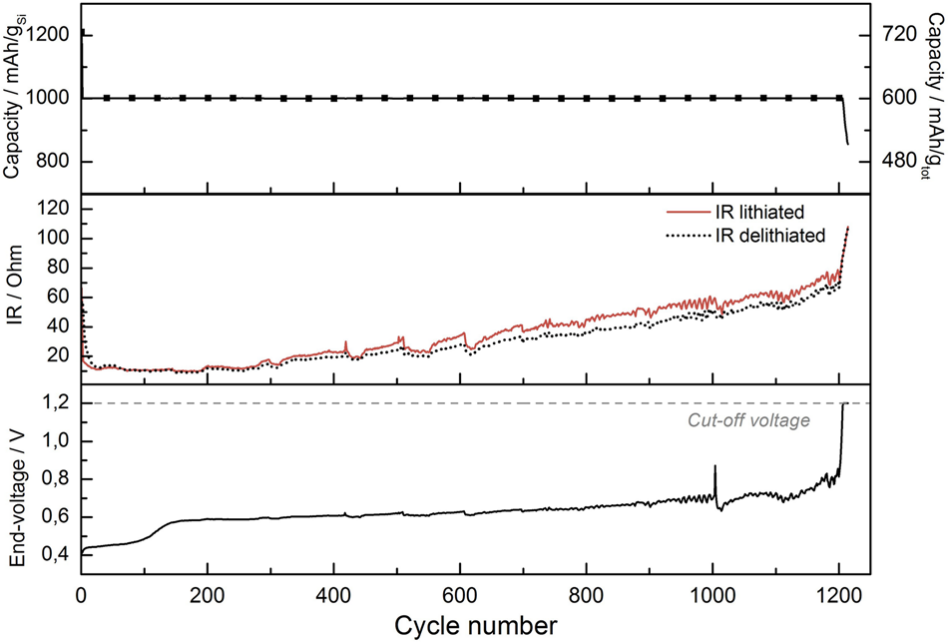
Cycle performance of Si/C composite with CMC&SBR binder with the use of 10% FEC as an electrolyte additive to LP30 under limited cycling at 1000 mAh g^−1^ of Si. The development of discharge capacity (top), internal resistance(middle) and the end-of-charge voltage (bottom) can be monitored as a function of cycle number.

**Figure 9:**
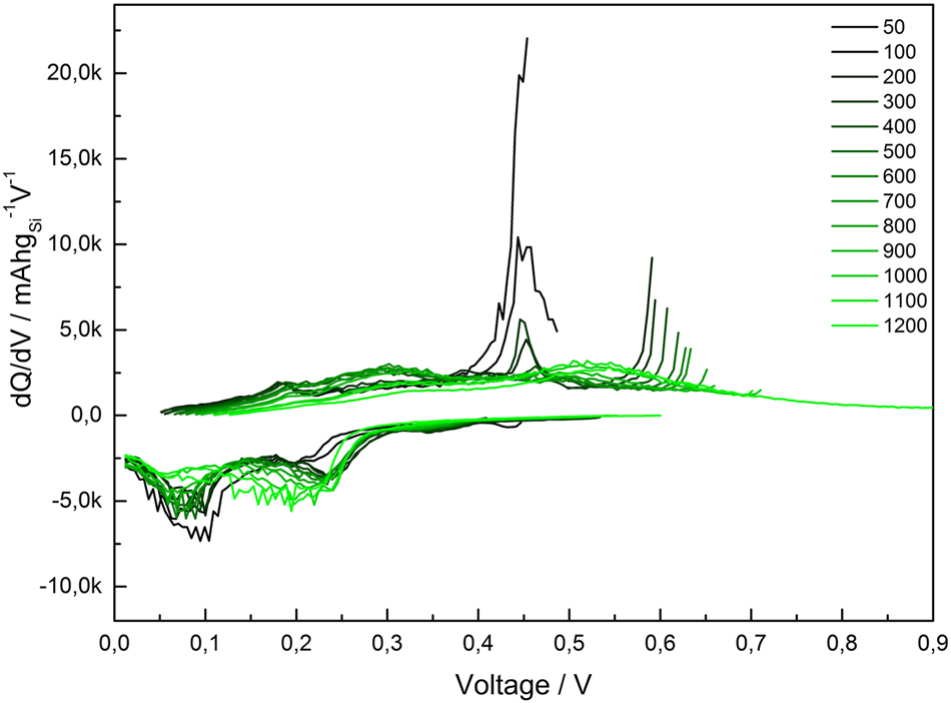
Differential capacity profiles from cycle 50 to 1200 of Si/C composite with CMC&SBR binder with the use of 10% FEC as an electrolyte additive to LP30 under limited cycling at 1000 mAh g^−1^ of Si.

To test the stated beneficial effects of electrolyte additives, cells were prepared with 10 wt% FEC added to the LP30 electrolyte and cycled with a limited capacity regime with a cutoff at 1000 mAh g^−1^ Si at C/5-rate. One must be careful when implementing a limited capacity regime, as the observable capacity degradation is masked due to a surplus of available lithium in the cells. Only once the reservoirs are depleted, a sudden cell failure occurs. However, a limited capacity regime simulates very well the conditions the anode will experience during full cell operation as the anode in a commercial cell will be dimensioned to hold more lithium than what is needed at the cathode. To have some indication of the degradation of the cell during limited capacity cycling, it is possible to measure both the internal resistance of the cell as well as the end-voltage as function of cycle number. The addition of FEC to the electrolyte creates different surface film components, consisting of flexible polycarbonates. Due to FEC decomposing at a higher voltage (1.4 V vs. Li/Li^+^) than the remaining electrolyte components, this flexible and more robust film is formed early on in the first cycle and provides the composite system with a higher stability^13^. The flexible polycarbonates have a better ability to accommodate the volume variations of the Si phase and can limit the contact between the electrode and the electrolyte. This will reduce the amount of SEI products precipitating and accumulating on the electrode at each cycle^39^. Figure 8 shows the cycle performance of a composite electrode with SBR/CMC binder and FEC as an electrolyte additive. In addition, the internal resistance of the cell and the end-voltage development are shown. At the set capacity of 1000 mAh g^−1^ Si (600 mAh g^−1^ of total electrode), the cell was able to keep the capacity for over 1200 cycles. Observing only the top graph, the cycle performance, one does not learn much about the changes in the electrode or the possible degradation mechanisms that are slowly induced on the electrode. But also examining the internal resistance, measured both in lithiated state and delithiated state, as well as observing the end-voltage increasing towards the cut-off voltage at 1.2 V, the sudden decrease in capacity in the top graph seems obvious. The internal resistance is seen to increase steadily, both in lithiated and delithiated state. The initial resistance starts at around 60 Ω, is then reduced to less than 20 Ω after a few cycles. This decrease is consistent with reduction of the non-conductive oxide film initially present on the surface of silicon. After 200 cycles, the internal resistance starts to increase and reaches a level of 100 Ω after the 1200 cycles. This can likely be attributed to cracking of the electrode, thickness increase and clogging of the electrode voids, which are all signals of the overall degradation mechanisms of the electrode. The end-voltage (lower figure) starts at 0.4 V and increases over the 1200 cycles to the set cut-off voltage at 1.2 V. However, distinct plateaus are observed at 0.45 V and at 0.6 V, where the first plateau is held for 150 cycles, while the plateau at 0.6 is held almost throughout the cycling until the sudden increase in end-voltage is observed. These plateaus are consistent with the differential capacity plots in Fig. 9, where we clearly observe large de-lithiation peaks around 0.45 V and 0.6 V. These peaks seem to be “consumed” (decrease in intensity) with increasing cycle number and coincides with utilization of surplus lithium left in the structure from the initial cycle. The high-end voltage around 1000 cycles indicates that the lithium left in the anode is now most likely very close to being completely depleted. After 1000 cycles, some irregular behavior is observed for the end-voltage, as it is slowly decreasing, and a “healing” of the electrode takes place. This “healing” could be due to solid state lithium diffusion from the edges within the electrode as the electrode was in a rest period at this stage. The deep de-lithiation at cycle 1000 could also have un-clogged previously clogged electrode voids (or otherwise exposed new area for electrochemical activity), which could explain the prolonged cycle life to around 1200 cycles.

### Increasing the capacity

**Figure 10:**
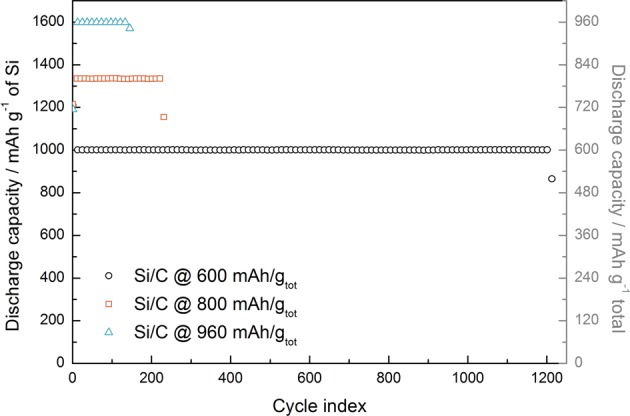
Cycle performance of electrodes cycled with various limited capacity.

As we have seen in the previous results, limited cycling simply allows for lithium reservoir distributed as the cell is cycled. This effect is clearly seen by increasing the capacity of the limited cycling as in Fig. 10. Here we see how the cycle life is significantly decreased as we increase the limiting cycling capacity. There are pros and cons with limited cycling, and it is important to be aware of the cons when implementing these kind of testing routines, such as “false” stability throughout. The pros are that one can simulate how a real cell would function until cell failure and relive some of the internal stress upon lithiation/delithiation of silicon, which would most likely improve cycle life as well. However, it is important to keep in mind that these are half cells, and therefore has a large surplus of available lithium. In a real cell the supplied lithium from the cathode would be limited also, and in further work it would be important use full cells systems when using limited capacity cycling.

## Conclusion

During half-cell testing, Silgrain® behaves as well as any other tested silicon material. In formative cycling, the material shows a signature more like amorphous or heavily ball milled silicon than for crystalline silicon. Mechanical processing methods reduced particle size according to XRD/TEM data, however no significant benefit of initial or long-term capacity was found by decreasing the particle size as the initial samples degraded quite fast (after only 10 cycles). To improve the tested silicon material- the following methods were used and evaluated; buffer solutions, binder composition, electrolyte additives and constrained cycling method. It was found that the use of buffer in the slurry significantly improved the long-term cycling of the silicon anode and silicon -carbon composite electrodes more than doubling the cycle life of the initially tested material. In addition, bonding properties of different binder materials was investigated. The use of CMC binder coupled with SBR showed improved stability compared to only using CMC. The properties of CMC (water solubility and thickening agent) and SBR (high flexibility and binding force) combined seem to complement each other resulting in a promising binder composition for silicon anodes. Finally, the influence of electrolyte additive, FEC, and limited cycling of in a CMC/SBR binder system resulted in over 1200 cycles with a capacity of 1000 mAh g^−1^ of Si. This constrained cycling method to control the silicon expansion seems to be very beneficial for long term performance. However, this limited capacity cycling has some challenges with regards to the observed capacity degradation as the real losses are somewhat hidden until sudden failure. Limited cycling simply allows for a lithium reservoir, which is clearly seen as the cycle life decreases once we increase the capacity limit above 1000 mAh g^−1^ of Si. By utilizing dQ/dV, end-voltage and internal resistance measurements, the actual degradation of the cell can be more accurately evaluated. For real cell simulation it is important to implement full cells as well to limit the available lithium to real levels.

## Data Availability

The datasets generated during and/or analyzed during the current study are available from the corresponding author on reasonable request.

## References

[1] Obrovac MN, Christensen L (2004). Structural changes in silicon anodes during lithium insertion/extraction. Electrochem Solid St.

[2] Tran TD, Feikert JH, Song X, Kinoshita K (1995). Commercial carbonaceous materials as lithium intercalation anodes. Journal of the Electrochemical Society.

[3] Huggins RA (1999). Lithium alloy negative electrodes. Journal of Power Sources.

[4] Liu, B. *et al*. Hierarchical silicon nanowires-carbon textiles matrix as a binder-free anode for high-performance advanced lithium-ion batteries. *Sci. Rep*. **3**, 10.1038/srep01622, http://www.nature.com/srep/2013/130408/srep01622/abs/srep01622.html#supplementary-information (2013). 10.1038/srep01622 PMC3621141 23572030

[5] Winter M, Besenhard JO (1999). Electrochemical lithiation of tin and tin-based intermetallics and composites. Electrochimica Acta.

[6] Bogart TD, Chockla AM, Korgel BA (2013). High capacity lithium ion battery anodes of silicon and germanium. Current Opinion in Chemical Engineering.

[7] Gonzalez J (2014). Three dimensional studies of particle failure in silicon based composite electrodes for lithium ion batteries. Journal of Power Sources.

[8] Wu H, Cui Y (2012). Designing nanostructured Si anodes for high energy lithium ion batteries. Nano Today.

[9] Yu B-C, Hwa Y, Kim J-H, Sohn H-J (2014). Characterizations and electrochemical behaviors of milled Si with a degree of amorphization and its composite for Li-ion batteries. Journal of Power Sources.

[10] Nguyen BPN, Chazelle S, Cerbelaud M, Porcher W, Lestriez B (2014). Manufacturing of industry-relevant silicon negative composite electrodes for lithium ion-cells. Journal of Power Sources.

[11] Chan CK, Ruffo R, Hong SS, Cui Y (2009). Surface chemistry and morphology of the solid electrolyte interphase on silicon nanowire lithium-ion battery anodes. Journal of Power Sources.

[12] Ulldemolins M (2012). Investigation on the part played by the solid electrolyte interphase on the electrochemical performances of the silicon electrode for lithium-ion batteries. Journal of Power Sources.

[13] Jaumann T (2015). SEI-component formation on sub 5 nm sized silicon nanoparticles in Li-ion batteries: the role of electrode preparation, FEC addition and binders. Physical Chemistry Chemical Physics.

[14] Wu H (2012). Stable cycling of double-walled silicon nanotube battery anodes through solid-electrolyte interphase control. Nat. Nanotechnol..

[15] Liu NA, Hu LB, McDowell MT, Jackson A, Cui Y (2011). Prelithiated Silicon Nanowires as an Anode for Lithium Ion Batteries. ACS Nano.

[16] Wang M-S, Song W-L, Wang J, Fan L-Z (2015). Highly uniform silicon nanoparticle/porous carbon nanofiber hybrids towards free-standing high-performance anodes for lithium-ion batteries. Carbon.

[17] Wang L (2010). A novel carbon-silicon composite nanofiber prepared via electrospinning as anode material for high energy-density lithium ion batteries. Journal of Power Sources.

[18] Park MS, Wang GX, Liu HK, Dou SX (2006). Electrochemical properties of Si thin film prepared by pulsed laser deposition for lithium ion micro-batteries. Electrochimica Acta.

[19] Kasavajjula U, Wang C, Appleby AJ (2007). Nano- and bulk-silicon-based insertion anodes for lithium-ion secondary cells. Journal of Power Sources.

[20] Kim JS (2015). Three-dimensional silicon/carbon core–shell electrode as an anode material for lithium-ion batteries. Journal of Power Sources.

[21] Li S (2014). Silicon/carbon composite microspheres with hierarchical core–shell structure as anode for lithium ion batteries. Electrochemistry Communications.

[22] Kim J-Y, Nguyen DT, Kang J-S, Song S-W (2015). Facile synthesis and stable cycling ability of hollow submicron silicon oxide–carbon composite anode material for Li-ion battery. Journal of Alloys and Compounds.

[23] Yao Y (2011). Interconnected Silicon Hollow Nanospheres for Lithium-Ion Battery Anodes with Long Cycle Life. Nano Letters.

[24] Szczech JR, Jin S (2011). Nanostructured silicon for high capacity lithium battery anodes. Energ Environ Sci.

[25] Lin D (2015). A high tap density secondary silicon particle anode fabricated by scalable mechanical pressing for lithium-ion batteries. Energ Environ Sci.

[26] Liu, N., Huo, K., McDowell, M. T., Zhao, J. & Cui, Y. Rice husks as a sustainable source of nanostructured silicon for high performance Li-ion battery anodes. *Sci. Rep*. **3**, 10.1038/srep01919, http://www.nature.com/srep/2013/130529/srep01919/abs/srep01919.html#supplementary-information (2013). 10.1038/srep01919 PMC3665957 23715238

[27] Reyter D (2013). An electrochemically roughened Cu current collector for Si-based electrode in Li-ion batteries. Journal of Power Sources.

[28] Profatilova IA, Stock C, Schmitz A, Passerini S, Winter M (2013). Enhanced thermal stability of a lithiated nano-silicon electrode by fluoroethylene carbonate and vinylene carbonate. Journal of Power Sources.

[29] Choi N-S (2006). Effect of fluoroethylene carbonate additive on interfacial properties of silicon thin-film electrode. Journal of Power Sources.

[30] Beattie SD (2016). Understanding capacity fade in silicon based electrodes for lithium-ion batteries using three electrode cells and upper cut-off voltage studies. Journal of Power Sources.

[31] Uchida S, Mihashi M, Yamagata M, Ishikawa M (2015). Electrochemical properties of non-nano-silicon negative electrodes prepared with a polyimide binder. Journal of Power Sources.

[32] Chakrapani V, Rusli F, Filler MA, Kohl PA (2012). Silicon nanowire anode: Improved battery life with capacity-limited cycling. Journal of Power Sources.

[33] Kam, K. C. & Doeff, M. M. Electrode Materials for Lithium Ion Batteries. *Material Matters* **7** (2012).

[34] Leblanc D (2015). Silicon as anode for high-energy lithium ion batteries: From molten ingot to nanoparticles. Journal of Power Sources.

[35] Gauthier M (2013). A low-cost and high performance ball-milled Si-based negative electrode for high-energy Li-ion batteries. Energ Environ Sci.

[36] Wang D, Gao M, Pan H, Wang J, Liu Y (2014). High performance amorphous-Si@SiOx/C composite anode materials for Li-ion batteries derived from ball-milling and *in situ* carbonization. Journal of Power Sources.

[37] Liu Z (2019). Silicon oxides: a promising family of anode materials for lithium-ion batteries. Chemical Society Reviews.

[38] Wang L, Xie X, Dinh KN, Yan Q, Ma J (2019). Synthesis, characterizations, and utilization of oxygen-deficient metal oxides for lithium/sodium-ion batteries and supercapacitors. Coordination Chemistry Reviews.

[39] Mazouzi D (2012). New insights into the silicon-based electrode’s irreversibility along cycle life through simple gravimetric method. Journal of Power Sources.

[40] Mazouzi D (2015). Critical roles of binders and formulation at multiscales of silicon-based composite electrodes. Journal of Power Sources.

[41] Bitsch B (2014). A novel slurry concept for the fabrication of lithium-ion battery electrodes with beneficial properties. Journal of Power Sources.

[42] Zhang R (2015). Water soluble styrene butadiene rubber and sodium carboxyl methyl cellulose binder for ZnFe_2_O_4_ anode electrodes in lithium ion batteries. Journal of Power Sources.

[43] Lim S, Kim S, Ahn KH, Lee SJ (2015). The effect of binders on the rheological properties and the microstructure formation of lithium-ion battery anode slurries. Journal of Power Sources.

